# Supplementary material for: Pyridoxine supplementation confers protection against SGPL1R222Q variant sphingosine phosphate lyase insufficiency syndrome
Source: bioRxiv. 2026 May 14:2026.05.11.724358. Preprint. [Version 1] doi: 10.64898/2026.05.11.724358 (PMC13192960; doi:10.64898/2026.05.11.724358)
Supplement: 1 [file NIHPP2026.05.11.724358V1-supplement-1.pdf]

bioRxiv preprint doi: https://doi.org/10.64898/2026.05.11.724358; this version posted May 14, 2026. The copyright holder has placed this preprint (which was not certified by peer review) in the Public Domain. It is no longer restricted by copyright. Anyone can legally share, reuse, remix, or adapt this material for any purpose without crediting the original authors.

**Table S1. Quantification of glomerulosclerosis**

| **Genotype** | **Total Glomeruli** | **Focal segmental glomerulosclerosis (%)** | **Global glomerulosclerosis (%)** |
|---|---|---|---|
| WT Hi-B6 (n = 3) | 241 | 0 | 0 |
| WT NA-B6 (n =3) | 175 | 0 | 0 |
| $SPL^{R222Q}$ Hi-B6 (n =3) | 230 | 0 | 0 |
| $SPL^{R222Q}$ NA-B6 (n =3) | 299 | 14.2 | 8.56 |

bioRxiv preprint doi: https://doi.org/10.64898/2026.05.11.724358; this version posted May 14, 2026. The copyright holder has placed this preprint (which was not certified by peer review) in the Public Domain. It is no longer restricted by copyright. Anyone can legally share, reuse, remix, or adapt this material for any purpose without crediting the original authors.

**Table S2. List of primers used for mutagenesis and qRT-PCR.**

| **Primer name** | **Sequence (5'-3')** |
|---|---|
| h-*SGPL1*-WT-F | GGAATTCCATATGAATAAAACTAAGGATGATATCAGCAAGAATATG |
| h-*SGPL1*-WT-R | ATATATCTCGAGTTAATGATGATGGTGATGATGACCGCTACCGTGAG GTT TAGGGCTTCCATTCATCTG |
| h-*SGPL1*- R222Q-F | AAAGCCTAC**CAG**GATCTTGCGTTTGAGAAGGG |
| h-*SGPL1*- R222Q-R | AACGCAAGATC**GTC**GTAGGCTTTGCATGCCAT |
| h-*SGPL1*- R222W-F | AAAGCCTAC**TGG**GATCTTGCGTTTGAGAAGGG |
| h-*SGPL1*- R222W-R | AACGCAAGATC**CCA**GTAGGCTTTGCATGCCAT |
| m*Lcn2*-F | TGGCCCTGAGTGTCATGTG |
| m*Lcn2*-R | CTCTTGTAGCTCATAGATGGTGC |
| m*Timp1*-F | GCAACTCGGACCTGGTCATAA |
| m*Timp1*-R | CGGCCCGTGATGAGAAACT |
| m*Socs1*-F | CTGCGGCTTCTATTGGGGAC |
| m*Socs1*-R | AAAAGGCAGTCGAAGGTCTCG |
| m*Socs3*-F | CCCTTGCAGTTCTAAGTTCAACA |
| m*Socs3*-R | ACCTTTGACAAGCGGACTCTC |
| m*Tnf-α*-F | CAGGCGGTGCCTATGTCTC |

bioRxiv preprint doi: https://doi.org/10.64898/2026.05.11.724358; this version posted May 14, 2026. The copyright holder has placed this preprint (which was not certified by peer review) in the Public Domain. It is no longer restricted by copyright. Anyone can legally share, reuse, remix, or adapt this material for any purpose without crediting the original authors.

| | |
|---|---|
| m*Tnf-α*-R | CGATCACCCCGAAGTTCAGTAG |
| m*Tgfβ*-F | CCACCTGCAAGACCATCGAC |
| m*Tgfβ*-R | CTGGCGAGCCTTAGTTTGGAC |
| m*Ccl2*-F | TGCCCTAAGGTCTTCAGCAC |
| m*Ccl2*-R | AAGGCATCACAGTCCGAGTC |
| m*Ccl1 7*-F | AAGCTCATCTGTGCAGACCC |
| m*Ccl1 7*-R | CAGGGACTTCTGCTCTGTGG |
| m*Cx3c r1*-F | GCCTGTTATTTGGGCGACAT |
| m*Cx3c r1*-R | ACAAAGAGCAGGTCGCTCAA |
| m*Tnfsf 15*-F | TGTGTGGTGACATCAGTCGG |
| m*Tnfsf 15*-R | TCTTCCAAGGAGAACGTGGC |
| m*Dcn*-F | CTTCCTTCTGGCACAAGTCT |
| m*Dcn*-R | CAGAACACTGCACCACTCGA |
| m*Ddr2*-F | GGCTGGACTCTGAAGAAGGA |
| m*Ddr2*-R | CCATCCCGACTGTAGTTGAT |
| m*Gre m2*-F | TGGTGGCTGTGCTGGTAAAG |
| m*Gre m2*-R | TTGAGGTACTTGCGCTCGGT |
| m*Osmr*-F | GGACACGAAGAGGTCAAGGA |
| m*Osmr*-R | ATCATCTGAGGTGATGGTGG |
| m*Papp a*-F | ACAGCCCTGTAAATCCAGTG |
| m*Papp a*-R | CACTAGAGTCCCAATCACTGG |

| | |
|---|---|
| m*Stc2*-F | CATCAAGGATGCCCTGAGGT |
| m*Stc2*-R | TCACACCGACGTTCTCCTGG |
| m-B-Actin-F | GGCTGTATTCCCCTCCATCG |
| m-B-Actin-R | CCAGTTGGTAACAATGCCATGT |
